# Primary Angle Closure Observed During a House Visit: A Case Treated With Laser Iridotomy

**DOI:** 10.7759/cureus.66321

**Published:** 2024-08-06

**Authors:** Hiroki Nishimura, Rohan J Khemukani, Ryota Yokoiwa, Shintaro Nakayama, Eisuke Shimizu

**Affiliations:** 1 Department of Research, OUI Inc., Tokyo, JPN; 2 Department of Ophthalmology, Keio University School of Medicine, Tokyo, JPN; 3 Department of Research, Yokohama Keiai Eye Clinic, Kanagawa, JPN

**Keywords:** smart eye camera, angle closure, glaucoma, laser iridotomy, gp

## Abstract

Laser iridotomy (LI) is an effective treatment for patients with pupillary block mechanisms. Here, we report a case of LI performed on a patient with primary angle closure (PAC) and elevated intraocular pressure (IOP), who was unsuitable for other treatments due to specific social circumstances. The patient, a 97-year-old female residing in a private nursing home, had a medical history notable only for mild dementia and was wheelchair-bound. She had not undergone ophthalmologic evaluation for over 50 years. The patient presented with intermittent tenderness and redness in the left eye. Therefore, an ophthalmologist visited the nursing home. Examination revealed visual acuity of 20/200 in the right eye and 20/100 in the left eye, IOP of 24 mmHg in the right eye and 26 mmHg in the left eye, no conjunctival hyperemia, shallow anterior chambers, and nuclear sclerosis grade 4 cataracts in both eyes. Fundus examination was challenging due to lens opacity, and both optic nerve papillae appeared pale. Given her history of episodic eye pain and hyperemia, PAC was diagnosed. Treatment options, including eye drops and cataract surgery, were discussed. However, the patient opted for LI due to her advanced age and inability to attend frequent follow-up visits. LI was successfully performed on both eyes during her visit to the clinic. One week post-procedure, IOP decreased to 12 mmHg bilaterally, with no complications. This case demonstrates that LI can be a viable option for managing PAC and high IOP in patients who are not candidates for surgery or eye drops due to social constraints.

## Introduction

Laser iridotomy (LI) is a therapeutic procedure designed to alleviate pupillary block, equalize pressure between the anterior and posterior chambers, and widen the anterior chamber angle [1]. It is indicated for conditions such as primary angle closure (PAC) and primary angle closure glaucoma (PACG) and is also applicable for managing primary angle closure caused by pupillary block, primary and secondary angle closure glaucoma, narrow-angle eyes requiring prevention of relative pupillary block, and plateau iris configurations [2].

In Japan, the rapidly aging population has led to an increased demand for ophthalmic care within home settings. As the elderly population grows and disease trends shift, healthcare priorities have increasingly focused on improving patients' quality of life through comprehensive care approaches. While home medical care is typically provided by internists, primary care physicians, and visiting nurses, the specialized nature of ophthalmic treatment has limited its implementation in home care settings [3]. General practitioners (GPs) play a pivotal role in the initial assessment and triage of patients with potential ophthalmic conditions. Their ability to recognize early signs and symptoms can significantly impact patient outcomes through prompt referral to ophthalmology services. However, recent advancements in mobile diagnostic equipment have facilitated the delivery of comprehensive ophthalmic care not only in clinics but also within home care environments and through doctor-to-doctor telemedicine models [4].

This report presents a case in which LI was successfully performed during a home visit on a patient with PAC and elevated intraocular pressure. The patient's unique social circumstances rendered other treatment options unfeasible, highlighting the potential for LI as a viable home-based therapeutic intervention.

## Case presentation

A 97-year-old Japanese female resident of an assisted living facility was examined due to frequent episodes of unilateral eye redness and pain reported by the patient and her family over the past few months. At the time of examination, the patient exhibited no significant symptoms. Despite mild cognitive decline, she was able to move with the aid of a wheelchair and maintain a seated position. She had been residing in the facility for eight years and had not visited other medical institutions, relying solely on home medical care.

The patient had no significant ophthalmic history, having not visited an ophthalmologist for over 50 years. Her systemic medical history included hypertension and a post-surgical status for endometrial cancer. Medications prescribed by the home care physician included amlodipine besylate 5 mg and 0.1% hyaluronic acid eye drops.

**Figure 1:**
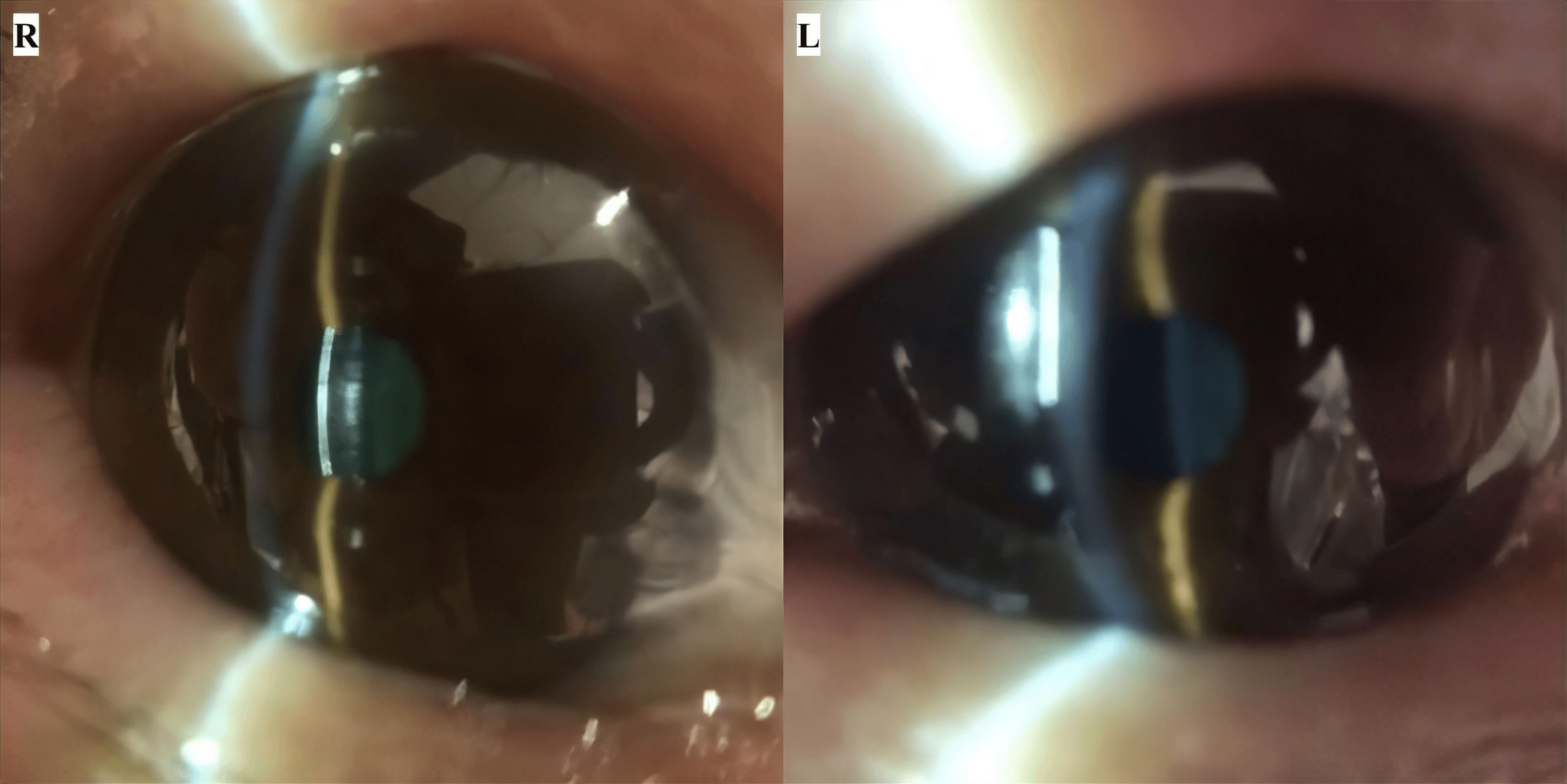
Anterior segment findings at initial home care (day 0) Anterior segment images captured by a portable slit-lamp device and a smart eye camera show dermatochalasis of both eyelids, shallow anterior chambers in both eyes, and nuclear sclerosis grade 4 cataracts.

Initial findings during the home visit included unmeasurable visual acuity and intraocular pressures of 23.0/24.0 mmHg (right/left) using iCare tonometry. Examination of the anterior segment using a portable slit-lamp device and a smart eye camera (SEC) revealed dermatochalasis of both eyelids, shallow anterior chambers in both eyes, no conjunctival hyperemia, and no corneal edema or clouding. Both lenses exhibited nuclear sclerosis (NS) grade 4 cataracts. Fundus examination revealed optic nerve head cupping and pallor in both eyes, but further detailed observations were challenging. Due to the limited diagnostic tools available during the home visit, the patient was referred to the clinic for further examination, considering her activities of daily living (ADLs) (Figure 1, Table 1).

**Table 1:** Intraocular pressure trends High intraocular pressure decreased to the normal range after laser iridotomy.

Day	Examination at	Right eye	Left eye	Measurement tools
0	Initial home care	23.0 mmHg	24.0 mmHg	iCare
20	Clinic	24.0 mmHg	26.0 mmHg	Applanation tonometer
40	Home care post-laser	15.0 mmHg	17.0 mmHg	iCare
74	Home care post-laser	13.0 mmHg	14.0 mmHg	iCare
102	Home care post-laser	15.0 mmHg	15.0 mmHg	iCare

**Figure 2:**
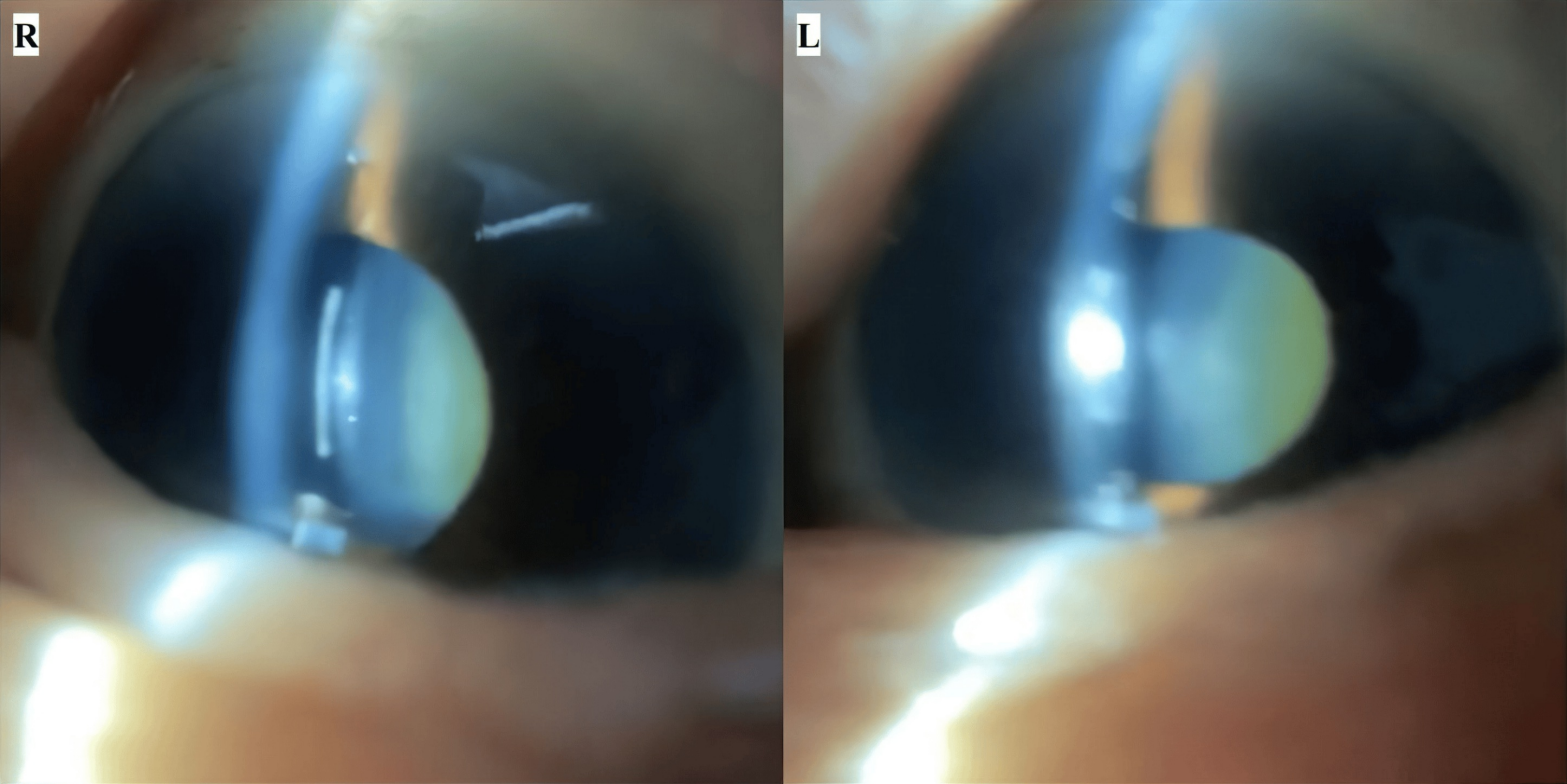
Anterior segment findings at the clinic (day 20) Anterior segment images were captured using a portable slit-lamp device and a smart eye camera. Similar to the initial examination, dermatochalasis of both eyelids, shallow anterior chambers in both eyes, and nuclear sclerosis grade 4 cataracts in both lenses were observed.

On the 20th day, the patient visited the clinic. Physical examination at the clinic revealed visual acuities of RV = (20/200 x S+4.00D:C-2.75Ax90) and LV = (20/100 x S+1.50D:C-4.00Ax70), and intraocular pressure (IOP) of 24.0/26.0 mmHg (noncontact tonometry). Anterior segment findings were consistent with the home visit, showing shallow anterior chambers and NS grade 4 cataracts without corneal or conjunctival abnormalities (Figure 2, Table 1).

**Figure 3:**
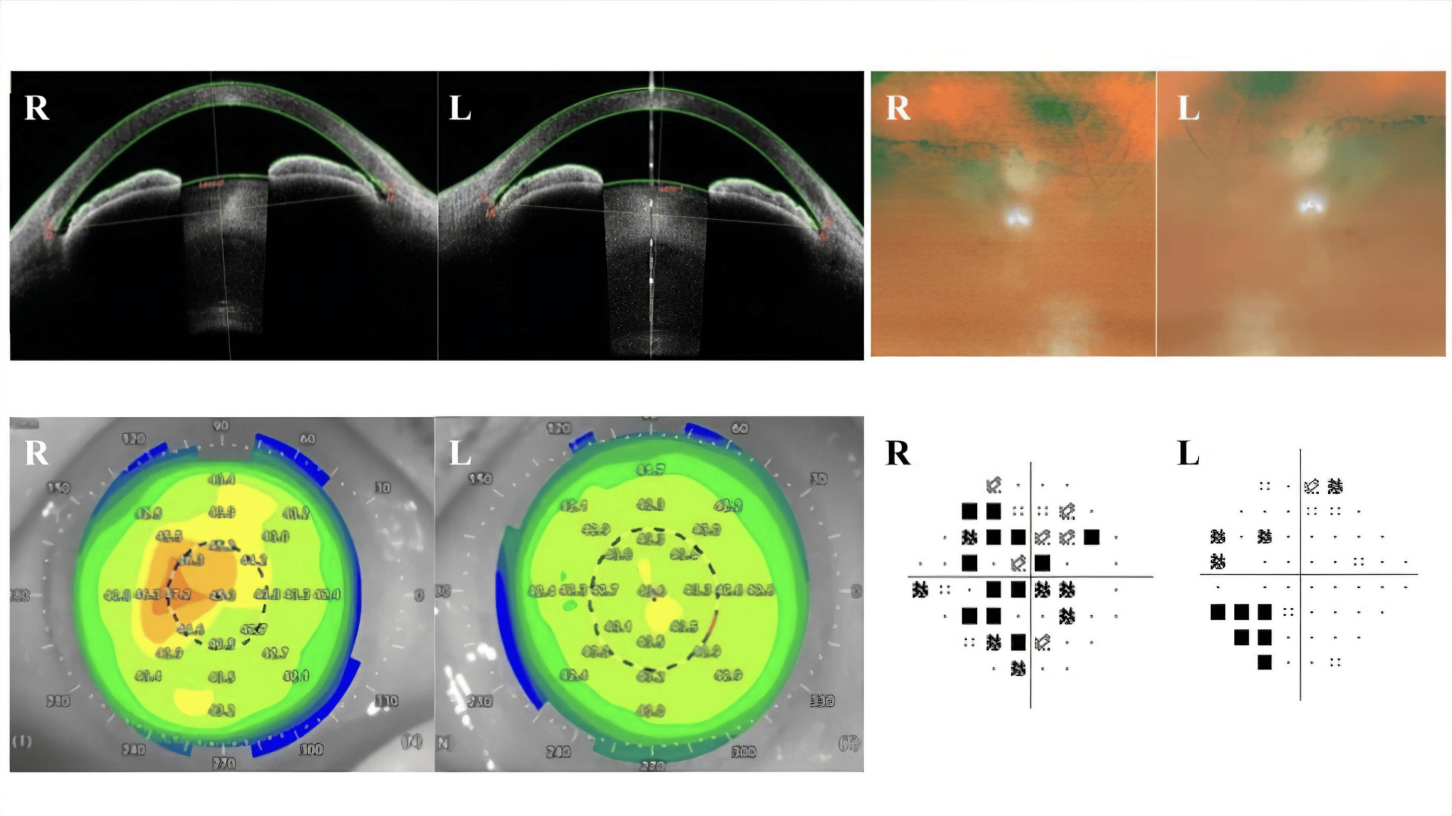
Other findings at the clinic This figure presents multiple diagnostic images and measurements from a comprehensive eye examination at the clinic. Anterior chamber depth: 1.83 mm (both eyes); TIA 0°: R 15.3°/L 20.6°, TIA 180°: R 18.4°/L 14.9°; corneal endothelial cell count: R 2,318/mm²/L 2,446/mm²; MD: -11.78/-10.07 dB; and PSD: 3.51/5.39 dB. TIA: Trabecular-iris angle; MD: Mean deviation; PSD: Pattern standard deviation; dB: Decibels.

Gonioscopy revealed angle closure in both eyes, classified as Grade II-III according to Scheie's classification, with no evident peripheral anterior synechiae. Anterior segment optical coherence tomography (OCT) showed central anterior chamber depths of 1.83 mm in both eyes, trabecular-iris angles of 15.3°/20.6° (TIA0° R/L) and 18.4°/14.9° (TIA180° R/L), and corneal endothelial cell counts of 2,318/2,446 cells/mm² (right/left). Fundus examination confirmed optic nerve cupping and pallor, but good-quality fundus images were not obtained due to cataracts. Visual field testing showed mean deviation (MD) values of -11.78/-10.07 dB (right/left), pattern standard deviation (PSD) values of 3.51/5.39 dB (right/left), and foveal threshold values of 19/24 dB (right/left), as shown in Figure 3.

Based on these findings, the patient was diagnosed with PACG (or PAC), cataracts, and elevated IOP in both eyes. Considering her cognitive decline and glaucoma severity, combined with her medical history and comorbidities, PACG was prioritized. Surgical intervention for cataract extraction was suggested due to her ADL allowing for clinic visits. However, the family expressed a preference for noninvasive treatment, and the facility indicated difficulties in managing eye drop administration (though oral medication could be managed). Consequently, LI was proposed and agreed upon for immediate treatment.

Bilateral LI was performed using an yttrium aluminum garnet (YAG) laser (YC-200, NIDEK, Aichi, Japan), creating a 100-200 μm opening in the superior temporal quadrant of both eyes. The procedure was completed without complications such as anterior chamber bleeding or postoperative IOP spikes. Follow-up was minimized due to financial constraints, with a prescription of acetazolamide and potassium L-aspartate for three days and follow-up via telemedicine using a handheld slit-lamp microscope and tonometry devices brought by a certified optometrist (CO).

**Figure 4:**
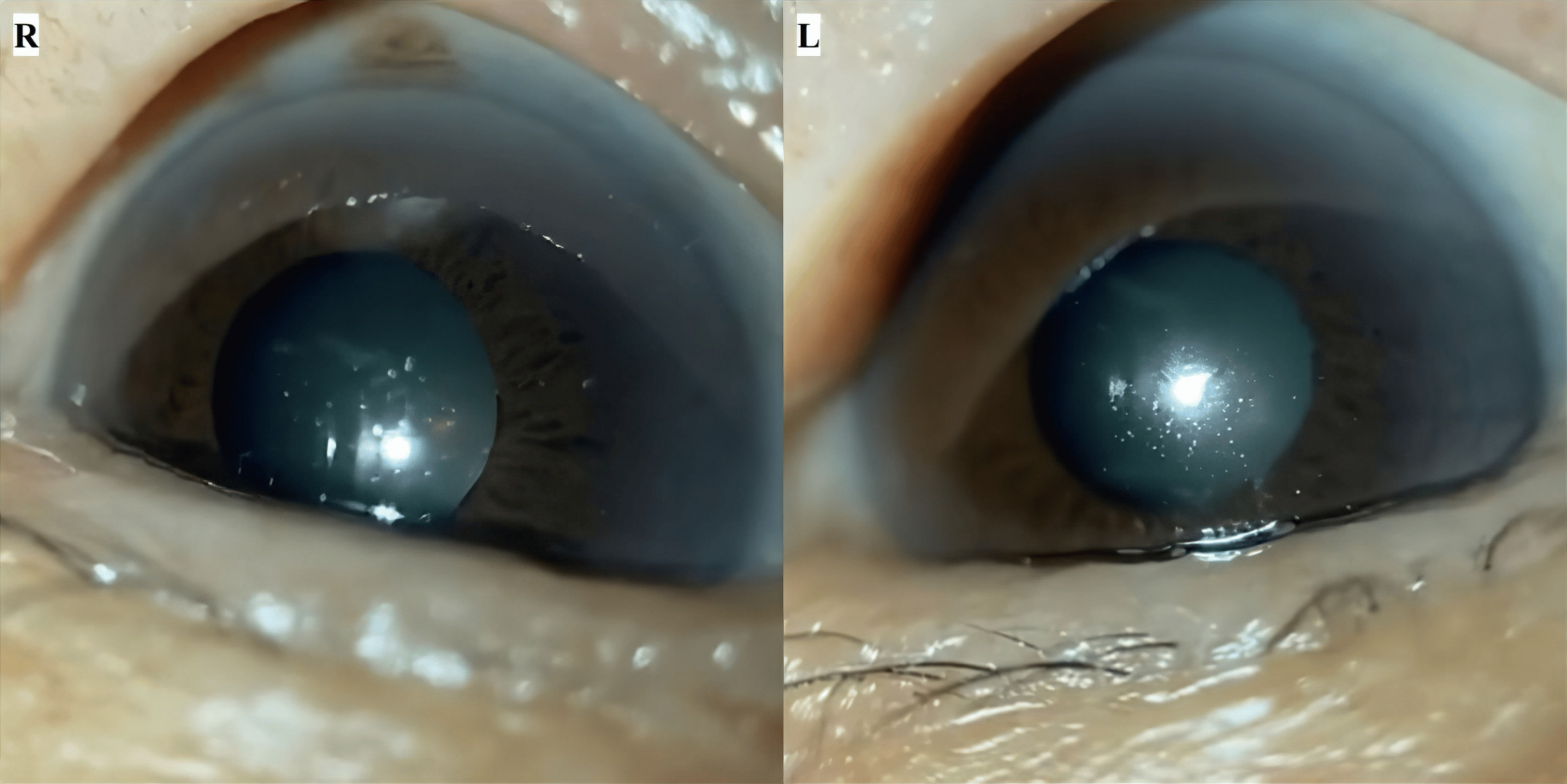
Anterior segment findings at home care by certified optometrist (day 40) Images and measurements represent findings during a home care visit on day 40, showing the patient's ocular status. Anterior segment images were captured by a portable slit-lamp device and a smart eye camera. No inflammation was observed in either anterior chamber.

On the 40th day, a CO visit revealed an IOP of 15.0/17.0 mmHg (right/left) using iCare tonometry. Anterior segment findings were unchanged, with no conjunctival or corneal abnormalities and patent LI openings in the superior temporal iris of both eyes. The cataracts remained unchanged. Based on these findings, the patient was considered to be in good condition, and monthly follow-up was recommended (Figure 4, Table 1).

Further CO visits on the 74th and 102nd days revealed IOP of 13.0/14.0 mmHg and 15.0/15.0 mmHg (right/left), respectively, with no changes in anterior segment findings, indicating a stable course (Table 1).

## Discussion

In this case, during a home visit by an ophthalmologist, PAC and elevated IOP were observed, leading to a diagnosis of PACG. Given the patient's social background, LI was performed on both eyes. The presence of symptoms such as eye pain and a history of recurrent conjunctival hyperemia, along with various findings, strongly suggested an angle closure mechanism. Treatment decisions were made collaboratively among the physician, the patient, and the family, considering not only the disease itself but also the patient's social background and economic constraints. This approach successfully achieved intraocular pressure control. However, the absence of a baseline and poor adherence to prescribed eye drops, critical components of standard glaucoma management, posed challenges in selecting treatment options aimed at lowering intraocular pressure and preserving the remaining visual field [5].

The prevalence of ophthalmic diseases in home care patients is reported to be 60%, with more than 20% of cases not identified by the primary care physician [6]. This highlights that ophthalmic conditions are often neglected in home care settings. In fact, the uptake of eye care services for the elderly in residential care remains poor despite the provision of services for free [7]. This case exemplifies such neglect, and if left unnoticed and untreated, the patient's quality of life could have significantly deteriorated, underscoring the growing importance of ophthalmic intervention in home care cases.

Improving adherence requires cooperation from patients, their families, and care facilities. While there is evidence supporting the enhancement of treatment outcomes through proper guidance and management systems for patients who can visit clinics, there is a lack of evidence for home-bound or remote patients. Therefore, cooperation among healthcare providers, caregivers, and patients becomes even more crucial [8].

In this case, due to social background, postoperative follow-up was conducted via telemedicine using visits by a CO instead of a physician. The CO utilized portable ophthalmic equipment for examinations, which can make an anterior segment diagnosis the same as a conventional slit-lamp microscope, with diagnoses provided remotely by an ophthalmologist [9-16].

Given the limited prevalence of ophthalmic care in home settings, the demand for telemedicine using portable ophthalmic equipment is expected to increase [17]. However, this could potentially lead to an increased workload for visiting physicians and ophthalmologists, necessitating efficient task shifting and sharing with paramedical staff.

## Conclusions

In this case, LI was performed on a patient with PAC and elevated IOP, where both surgical and eye drop treatments were challenging due to social circumstances. This intervention resulted in a favorable IOP outcome. While regular patient visits are essential for effective glaucoma management, it is anticipated that Japan will encounter an increase in the number of cases where such visits are difficult. Consequently, there will be a growing demand for equipment that enables glaucoma management in elderly care facilities and a need for task shifting and sharing of medical duties among paramedical staff.

## References

[1] Lee JW, Lee JH, Lee KW (2009). Prognostic factors for the success of laser iridotomy for acute primary angle closure glaucoma. Korean J Ophthalmol.

[2] Radhakrishnan S, Chen PP, Junk AK, Nouri-Mahdavi K, Chen TC (2018). Laser peripheral iridotomy in primary angle closure: a report by the American Academy of Ophthalmology. Ophthalmology.

[3] Yusufu M, Bukhari J, Yu X, Lin TP, Lam DS, Wang N (2021). Challenges in eye care in the Asia-Pacific region. Asia Pac J Ophthalmol (Phila).

[4] Ong J, Tan G, Ang M, Chhablani J (2022). Digital advancements in retinal models of care in the post-COVID-19 lockdown era. Asia Pac J Ophthalmol (Phila).

[5] Quaranta L, Novella A, Tettamanti M, Pasina L, Weinreb RN, Nobili A (2023). Adherence and persistence to medical therapy in glaucoma: an overview. Ophthalmol Ther.

[6] Panagioti M, Khan K, Keers RN (2019). Prevalence, severity, and nature of preventable patient harm across medical care settings: systematic review and meta-analysis. BMJ.

[7] Marmamula S, Kumbham TR, Modepalli SB, Chakrabarti S, Keeffe JE (2023). Barriers to uptake of referral eye care services among the elderly in residential care: the Hyderabad Ocular Morbidity in Elderly Study (HOMES). Br J Ophthalmol.

[8] Waterman H, Evans JR, Gray TA, Henson D, Harper R (2013). Interventions for improving adherence to ocular hypotensive therapy. Cochrane Database Syst Rev.

[9] Shimizu E, Ogawa Y, Yazu H (2019). "Smart eye camera": an innovative technique to evaluate tear film breakup time in a murine dry eye disease model. PLoS One.

[10] Yazu H, Shimizu E, Okuyama S (2020). Evaluation of nuclear cataract with smartphone-attachable slit-lamp device. Diagnostics (Basel).

[11] Shimizu E, Yazu H, Aketa N (2021). A study validating the estimation of anterior chamber depth and iridocorneal angle with portable and non-portable slit-lamp microscopy. Sensors (Basel).

[12] Yazu H, Shimizu E, Sato S (2021). Clinical observation of allergic conjunctival diseases with portable and recordable slit-lamp device. Diagnostics (Basel).

[13] Shimizu E, Yazu H, Aketa N (2021). Smart eye camera: a validation study for evaluating the tear film breakup time in human subjects. Transl Vis Sci Technol.

[14] Handayani AT, Valentina C, Suryaningrum IG (2023). Interobserver reliability of tear break-up time examination using “smart eye camera” in Indonesian remote area. Clin Ophthalmol.

[15] Andhare P, Ramasamy K, Ramesh R, Shimizu E, Nakayama S, Gandhi P (2023). A study establishing sensitivity and accuracy of smartphone photography in ophthalmologic community outreach programs: review of a smart eye camera. Indian J Ophthalmol.

[16] Borselli M, Toro MD, Rossi C (2024). Feasibility of tear meniscus height measurements obtained with a smartphone-attachable portable device and agreement of the results with standard slit lamp examination. Diagnostics (Basel).

[17] Marx M, Wolf D, Pheng L (1991). Eye care in a nursing home. J Vis Impair Blind.

